# Bilateral Carotid-cavernous Fistulas Treated with Partial Embolization and Radiosurgery

**DOI:** 10.7759/cureus.5886

**Published:** 2019-10-10

**Authors:** Robert G Briggs, Phillip A Bonney, Ozer Algan, Anil D Patel, Michael E Sughrue

**Affiliations:** 1 Neurosurgery, University of Oklahoma Health Sciences Center, Oklahoma City, USA; 2 Nerosurgery, University of Southern California Keck School of Medicine, Los Angeles, USA; 3 Radiation Oncology, University of Oklahoma Health Science Center, Oklahoma City, USA; 4 Ophthalmology, Dean McGee Eye Institute, University of Oklahoma Health Science Center, Oklahoma City, USA; 5 Nerosurgery, Prince of Wales Private Hospital, Sydney, AUS

**Keywords:** carotid-cavernous fistula, radiosurgery, embolization, dural arteriovenous fistula

## Abstract

Bilateral carotid-cavernous fistulas (CCFs) are rare. In this paper, we report the case of an 88-year-old woman who presented with a two-month history of worsening visual symptoms and was subsequently found to have bilateral Barrow grade D CCFs. Cannulation and complete embolization of the offending vessels during angiography proved unsuccessful, and so the patient underwent adjuvant radiosurgery as salvage therapy with a good clinical outcome. This case adds to the limited but growing literature on the multi-modal management of CCFs.

## Introduction

Carotid-cavernous fistulas (CCFs) are abnormal connections between the carotid arterial circulation and the cavernous sinus venous drainage. The venous hypertension and congestion that develop are responsible for the typical presenting symptoms of diplopia, decreased visual acuity, proptosis, and chemosis seen in patients. Most fistulas are unilateral, though infrequently they can occur bilaterally. CCFs are often classified in anatomic terms, delineated in reference to their arterial source. A direct CCF refers to a direct connection with the internal carotid artery (ICA). An indirect CCF refers to a connection with a branching artery of either the ICA or external carotid artery (ECA). Direct CCFs, representing the vast majority of these fistulas, are most often caused by trauma, while indirect CCFs are thought to occur spontaneously [1]. Treatment of CCFs can usually be accomplished with endovascular surgery, with at least 80% of patients being cured with either a transarterial or transvenous approach [2]. However, in cases of failed intervention, other treatment modalities may need to be considered. In this report, we present the case of a patient with bilateral CCFs treated with radiosurgery following unsuccessful endovascular embolization. We subsequently discuss the role of radiosurgery in the management of CCFs.

## Case presentation

An 88-year-old woman with a past medical history of congestive heart failure, for which she received a pacemaker, presented to her primary care doctor with a two-month history of progressive double vision, right eye swelling, and left eyelid droop. She was referred to a neuro-ophthalmologist, whose examination revealed right proptosis with severe chemosis, complete left ptosis, a left third nerve palsy with pupillary involvement, and a right sixth nerve palsy. Otherwise, the patient had normal visual acuity and no evidence of optic neuropathy. CT angiogram was suggestive of bilateral CCF. She was subsequently referred to a neurointerventionalist for transarterial embolization.

**Figure 1:**
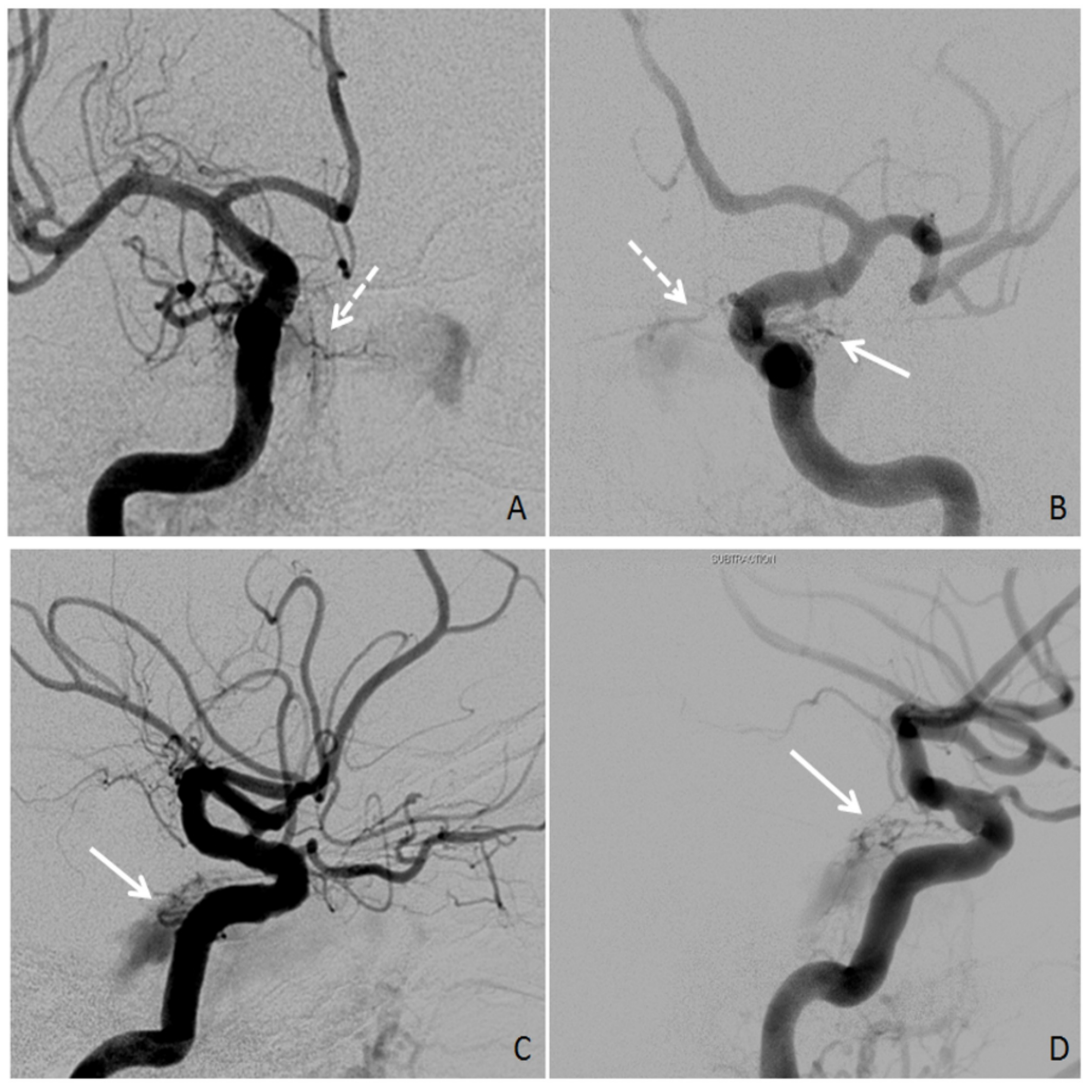
Bilateral Internal Carotid Artery Angiography Angiography showing internal carotid artery (ICA) fistulas (solid arrows: inferolateral trunk, dotted arrows: meningohypophyseal trunk). (A, B) Right and left ICA angiography, AP views. (C, D) Right and left ICA angiography, lateral views.

During the procedure, angiography first demonstrated a right-sided CCF, with contributions from both the right meningohypophyseal trunk (MHT) and right inferolateral trunk (ILT) (Figure 1A, 1C). Backflow into the right ECA was seen from multiple branches of the internal maxillary artery (IMA). Unfortunately, attempts to cannulate the MHT and ILT on the right were unsuccessful. Angiography of the left ICA revealed fistulas involving the vidian artery, left MHT, and left ILT (Figure 1B, 1D). Left IMA involvement was determined by way of backflow into the left ECA. Attempts to cannulate these small left-sided ICA branches also proved unsuccessful. As a result, the procedure was terminated without embolizing any vessels contributing to the bilateral Barrow grade D fistulas (Table 1).

**Table 1:** Barrow Classification of Carotid-cavernous Fistulas CCF: carotid-cavernous fistula, ECA: external carotid artery, ICA: internal carotid artery

Grade	Type of Fistula
A	ICA to cavernous sinus (i.e., direct CCF)
B	ICA meningeal branch to cavernous sinus
C	ECA meningeal branch to cavernous sinus
D	ICA and ECA meningeal branches to cavernous sinus

Two weeks later, we attempted a transvenous approach to embolize the fistulas, first through the left ophthalmic vein, which was unsuccessful, and then through the left femoral vein. The catheter was passed into the right internal jugular vein. At this point during the operation, there was significant difficulty in advancing the catheter superiorly, and attempts to pass the catheter into the right petrosal sinus proved unsuccessful. Similarly, the catheter could not be advanced through the left internal jugular vein to access the left petrosal sinus. After failed transvenous approaches, the right ECA was cannulated. Right ECA angiography demonstrated the involvement of several small branches of the IMA, including the artery of the foramen rotundum and a sphenoid branch from the middle meningeal artery (MMA). Partial embolization of the distal right IMA with Onyx and a single coil was associated with a reduction in blood flow through the fistula.

Given that endovascular embolization had failed and that the patient was interested in pursuing further treatment, a meeting was held between members of the neurointervention, radiation oncology, and neurosurgery teams. The branches of the bilateral ICAs were deemed to be inaccessible via cannulation. Likewise, embolization via a transvenous approach would not be possible on either the right or left side due to occluded petrosal sinuses. Due to her age, medical comorbidities, and the complexity of her fistulas, open surgery would not be in the patient’s best interest. Thus, the team decided to attempt re-embolization of the right IMA and left IMA, followed by Gamma Knife radiosurgery to the sites of the fistulas.

**Figure 2:**
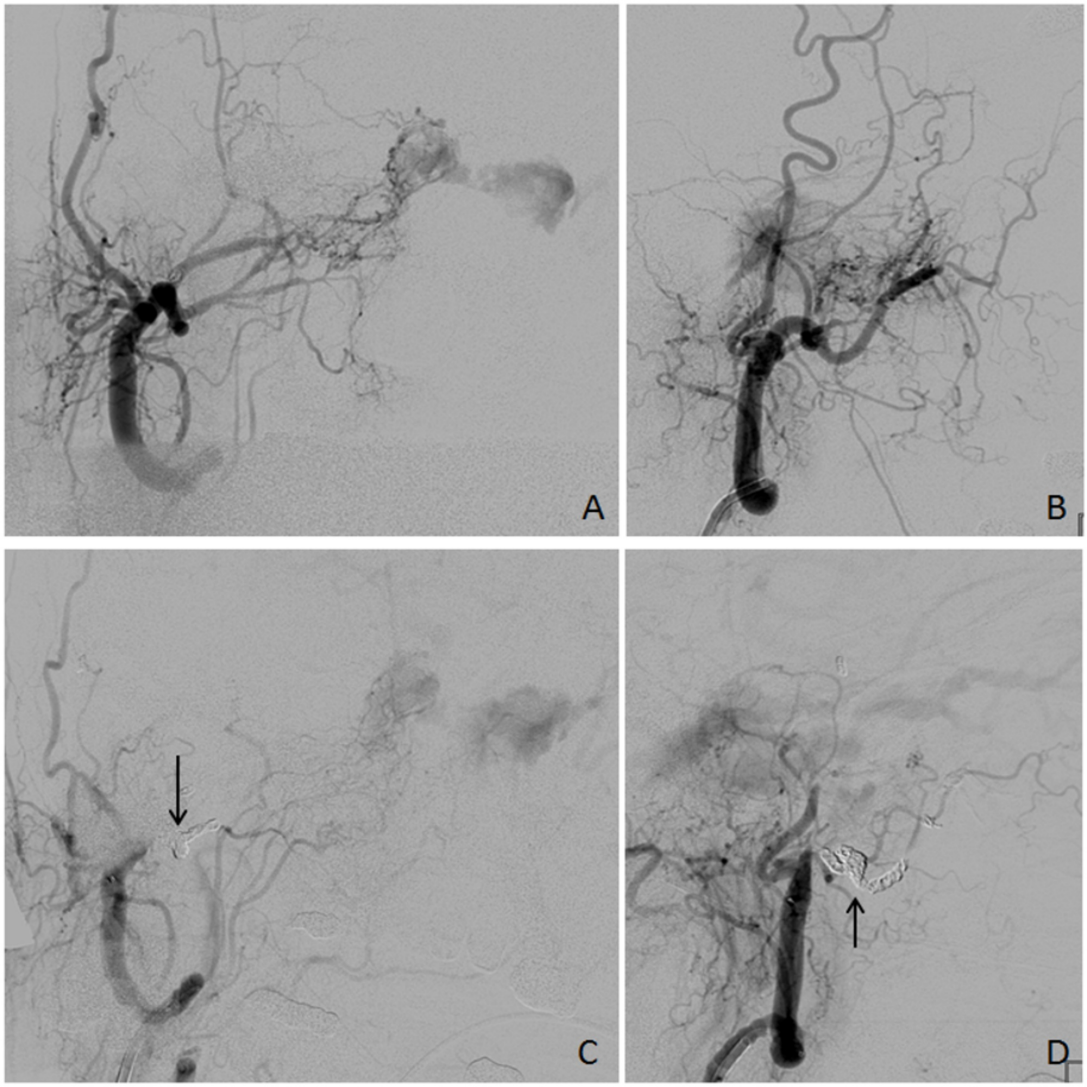
Right External Carotid Artery Angiography Right external carotid artery (ECA) angiography. (A, B) Before embolization, AP and lateral views, respectively. Multiple branches of the right ECA and internal maxillary artery are seen contributing to the carotid-cavernous fistula. (C, D) After embolization, AP and lateral views, respectively. Arrows indicate microcoils. Persistent flow is seen to the carotid-cavernous fistula from other ECA branches.

**Figure 3:**
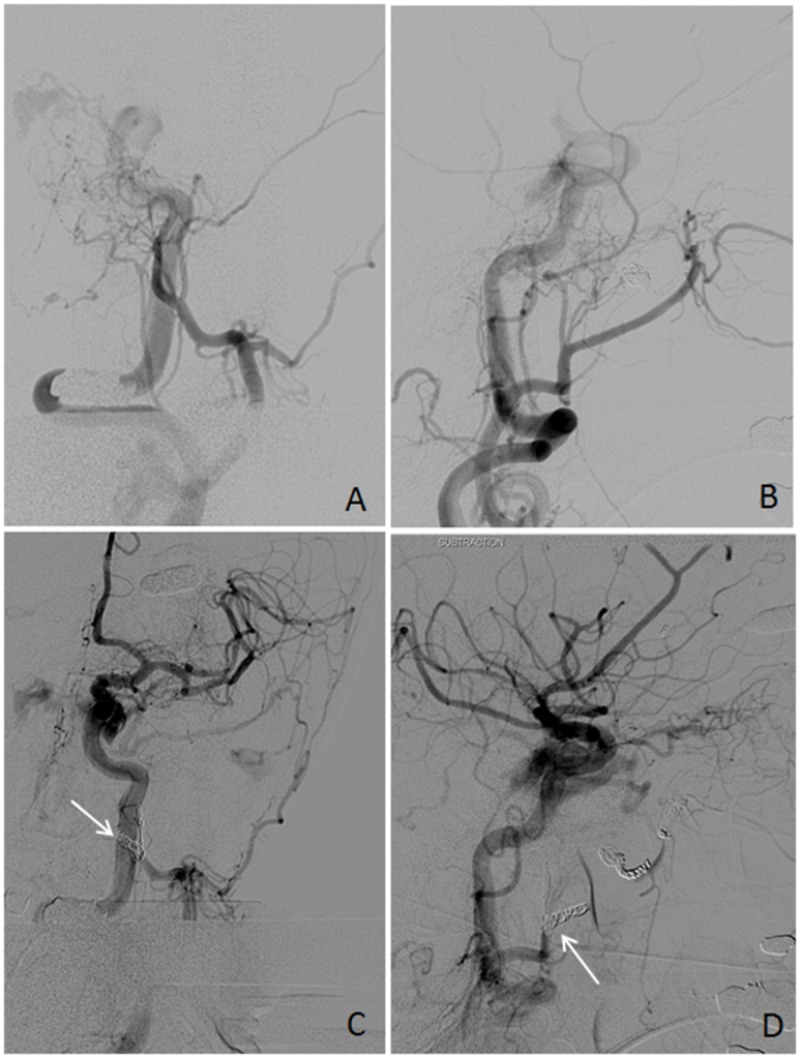
Left External Carotid Artery Angiography Left external carotid artery (ECA) angiography. (A, B) Before embolization, AP and lateral views, respectively. Multiple branches of left internal maxillary artery, including the middle meningeal artery, are seen contributing to the carotid-cavernous fistula. (C, D) After embolization, AP and lateral views, respectively. Arrows indicate coils. Reduced but persistent flow is seen flowing to the fistula.

Onyx and 10-microcoil embolization were performed on the distal right IMA, with significantly reduced flow into the cavernous sinus. Persistent contributions from other distal branches of the right ECA were noted, including from the ascending pharyngeal artery (Figure 2). On the left side, contribution from an occipital branch of the MMA was identified. Onyx embolization was performed on the left MMA without complication. In addition, branches of the distal left IMA were seen to contribute to the fistula network, so 6-microcoil embolization was performed on the distal left IMA with significantly reduced flow into the cavernous sinus (Figure 3).

**Figure 4:**
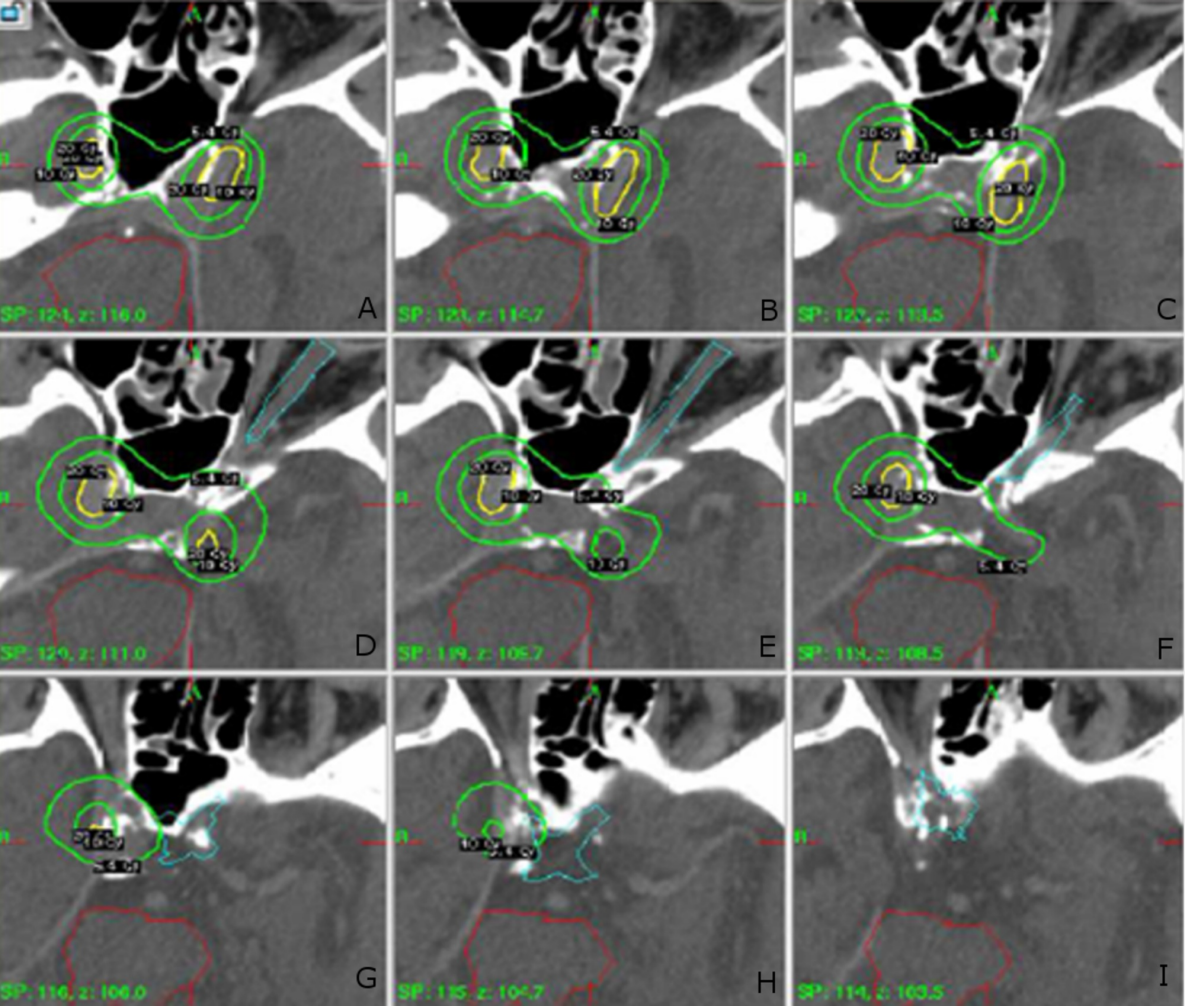
Gamma Knife Treatment Plan (A-I) CT scan with Gamma Knife treatment plan overlay. Outer green contour, 5.4 Gy. Inner green contour, 10 Gy. Yellow contour, 20 Gy. Turquoise contour, optic apparatus. Red contour, brainstem.

Directly following the third interventional procedure, Gamma Knife radiosurgery was performed. The fistulas were targeted with 20 Gy to the 50% isodense line bilaterally (Figure 4). A total of 11 shots were used. The maximum doses to the optic apparatus and brain stem were 5.7 Gy and 3.2 Gy, respectively. The patient tolerated both procedures well.

At first follow-up three weeks later, the right proptosis experienced by the patient had been dramatically improved, and she was able to open her left eye. At that time she continued to have intermittent diplopia with fluctuating visual acuity, though at additional follow-up, her visual symptoms continued to improve gradually. Her neuropathies have remained stable. Follow-up at 18 months demonstrated no new or worsening symptoms.

## Discussion

Options are limited for patients who fail endovascular therapy for indirect CCFs. Open surgery has been used historically. However, the morbidity of surgery often limits its use in elderly patients with significant medical comorbidities. As a result, radiosurgical treatment of indirect CCFs has been proposed as a safe, non-invasive adjunct or as primary therapy in patients who fail or cannot undergo endovascular surgery. A summary of the radiosurgery literature for CCFs is shown in Table 2, and it suggests that a multi-modal approach to CCFs may lead to improved outcomes in patients [3-17].

**Table 2:** Summary of Studies Using Radiosurgery or Radiotherapy for Indirect CCFs N: number of patients in the study, Gy: gray, Tx: treatment, Sx: symptom, RS: radiosurgery, RT: radiotherapy, NR: not recorded

Study	N	Bilateral	Tx	Max Dose (Gy)	Embolization	Obliteration	Chemosis and Proptosis Improvement	Cranial Nerve Palsy Improvement	Visual Acuity Improvement	Overall Sx Improvement
Rodrigues et al., 2014 [17]	1	NR	RS	NR	0	1/1 (100%)	NR	NR	NR	1/1 (100%)
Pan et al, 2010 [15]	41	13	RS	32	6 (15%)	37/41 (90%)	34/40 (85%)	10/14 (71%)	NR	NR
Onizuka et al., 2003 [14]	4	1	RS	29	1 (25%)	3/4 (75%)	3/3 (100%)	1/1 (100%)	NR	4/4 (100%)
Pollock et al., 1999 [13]	20	NR	RS	40	13 (65%)	13/15 (87%)	17/18 (94%)	10/13 (77%)	7/8 (88%)	NR
Hirai et al., 1998 [12]	18	3	RT	31	6 (33%)	3/7 (43%)	NR	NR	NR	15/18 (83%)
Guo et al., 1998 [11]	18	6	RS	28	0	12/15 (80%)	NR	NR	NR	NR
Hasuo et al., 1996 [10]	9	2	RT	30	9 (100%)	6/7 (86%)	NR	NR	NR	9/9 (100%)
Barcia-Salorio et al., 1994 [9]	22	NR	RS	30-40	0	20/22 (91%)	NR	NR	NR	NR
Pierot et al., 1992 [6]	1	0	RS	55	1 (100%)	1/1 (100%)	1/1 (100%)	1/1 (100%)	0	1/1 (100%)
Yasunaga et al., 1987 [5]	7	0	RS	31	1 (14%)	NR	NR	NR	NR	6/7 (8%)
Bitoh et al., 1982 [4]	2	NR	RS	31	0	0	1/1 (100%)	1/1 (100%)	0	2/2 (100%)

Repeat angiography is the gold standard for confirming obliteration of CCFs. However, magnetic resonance (MR) angiography has also been used for this purpose. Given our patient's early symptomatic improvement, age, and baseline functional status, she declined to undergo follow-up angiography in the endovascular suite. In addition, had the patient not had a pacemaker, MR angiography would have been performed.

## Conclusions

This case adds to the limited but growing literature on the multi-modal management of CCFs. For the patient presented in this report, partial embolization followed by radiosurgery for bilateral CCFs was associated with a good clinical outcome. The interventions were associated with little-to-no morbidity in an elderly patient with significant cardiac history.
